# Preoperative Ultrasonography Predicts Level II Lymph Node Metastasis in N1b Papillary Thyroid Carcinoma: Implications for Surgical Planning

**DOI:** 10.3390/biomedicines12071588

**Published:** 2024-07-17

**Authors:** Na Lae Eun, Jeong-Ah Kim, Yangkyu Lee, Ji Hyun Youk, Hyeok Jun Yun, Hojin Chang, Seok-Mo Kim, Yong Sang Lee, Hang-Seok Chang, Hyejin Yang, Soyoung Jeon, Eun Ju Son

**Affiliations:** 1 Department of Radiology, Thyroid Cancer Center, Gangnam Severance Hospital, Institute of Refractory Thyroid Cancer, Yonsei University College of Medicine, Gangnam-gu, Seoul 06273, Republic of Korea; enrlove@yuhs.ac (N.L.E.);; 2 Department of Pathology, Thyroid Cancer Center, Gangnam Severance Hospital, Institute of Refractory Thyroid Cancer, Yonsei University College of Medicine, Gangnam-gu, Seoul 06273, Republic of Korea; 3 Department of Surgery, Thyroid Cancer Center, Gangnam Severance Hospital, Institute of Refractory Thyroid Cancer, Yonsei University College of Medicine, Gangnam-gu, Seoul 06273, Republic of Korea; 4 Biostatistics Collaboration Unit, Yonsei University College of Medicine, Gangnam-gu, Seoul 06273, Republic of Korea

**Keywords:** thyroid cancer, papillary, lymph nodes, lymphatic metastasis, ultrasonography

## Abstract

Purpose: To investigate whether preoperative ultrasonographic (US) features of the index cancer and metastatic lymph nodes (LNs) are associated with level II LN metastasis in N1b papillary rmfthyroid carcinoma (PTC) patients. Materials and methods: We enrolled 517 patients (mean age, 42 [range, 6–80] years) who underwent total thyroidectomy and lateral compartment LN dissection between January 2009 and December 2015. We reviewed the clinicopathologic and US features of the index cancer and metastatic LNs in the lateral neck. Logistic regression analysis was performed to analyze features associated with level II LN metastasis. Results: Among the patients, 196 (37.9%) had level II metastasis on final pathology. In the preoperative model, larger tumor size (odds ratios [ORs], 1.031; 95% confidence interval [CI]: 1.011–1.051, *p* = 0.002), nonparallel tumor shape (OR, 1.963; 95% CI: 1.322–2.915, *p* = 0.001), multilevel LN involvement (OR, 1.906; 95% CI: 1.242–2.925, *p* = 0.003), and level III involvement (OR, 1.867; 95% CI: 1.223–2.850, *p* = 0.004), were independently associated with level II LN metastasis. In the postoperative model, non-conventional pathology remained a significant predictor for level II LN metastasis (OR, 1.951; 95% CI: 1.121–3.396; *p* = 0.018), alongside the presence of extrathyroidal extension (OR, 1.867; 95% CI: 1.060–3.331; *p* = 0.031), and higher LN ratio (OR, 1.057; 95% CI: 1.039–1.076; *p* < 0.001). Conclusions: Preoperative US features of the index tumor and LN may be helpful in guiding surgery in N1b PTC. These findings could enhance preoperative planning and decision-making, potentially reducing surgical morbidities by identifying those at higher risk of level II LN metastasis and tailoring surgical approaches accordingly.

## 1. Introduction

Papillary thyroid carcinoma (PTC) is the most common head and neck malignancy and generally has a favorable prognosis [1,2]. However, PTC frequently metastasizes to the central or lateral lymph nodes (LNs) in 30–60% of cases [3,4]. While regional LN metastasis is considered to have little impact on survival, recent studies indicate that lateral LN metastasis is associated with poorer prognosis and structural recurrence in PTC [5,6,7,8].

Therapeutic modified neck LN dissection is standard for N1b PTC, yet the extent of LN removal remains debated. Selective LN removal may be sufficient compared to comprehensive neck dissection (levels II to V) due to concerns over operative morbidity and functional outcomes, particularly with level IIb involvement [9,10]. Surgery in level IIb, near the spinal accessory nerve, can result in shoulder dysfunction in up to 27% of cases [9,11,12]. Given that the incidence of level IIb metastasis ranges from 2.1% to 22% [13,14,15,16,17,18,19,20,21,22], the necessity of routine comprehensive dissection and its impact on recurrence-free survival remain contentious.

Previous studies have focused on clinicopathologic factors for predicting level II metastasis [10,14,16,20,22,23,24,25,26]. However, the role of imaging features of the index thyroid cancer and LNs in predicting level II metastasis has not been thoroughly explored. Preoperative thyroid ultrasound (US) is a noninvasive and routine tool for clinical staging of PTC, offering potential insights into metastatic patterns. In light of these considerations, we hypothesized that preoperative US features could predict level II LN metastasis and aid in clinical decision-making for N1b PTC. This study aims to identify clinicopathologic and imaging features associated with level II LN metastasis in N1b PTC.

## 2. Materials and Methods

### 2.1. Study Population

**Figure 1:**
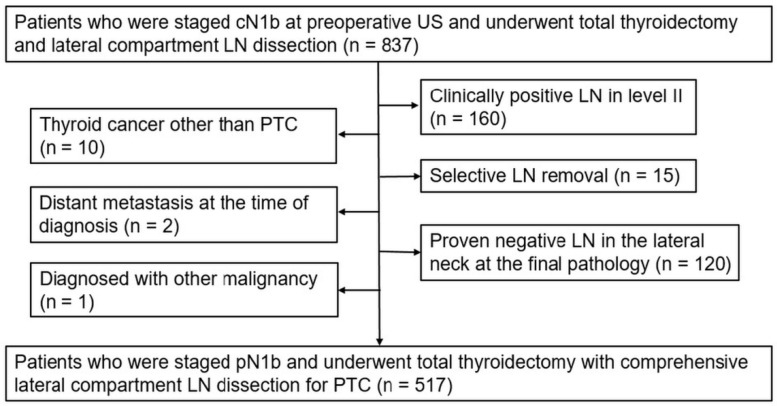
Flowchart of the study population. US, ultrasound; LN, lymph node; PTC, papillary thyroid carcinoma.

Our institutional review board approved this retrospective study and waived the requirement for informed consent. All patient information was handled in accordance with institutional guidelines to ensure confidentiality. Lateral neck LN metastasis (staged N1b) was noted in 837 patients on preoperative US performed between January 2009 and December 2015. Subsequently, the patients underwent total thyroidectomy and lateral LN dissection. We excluded patients with (a) clinically positive LNs in level II (*n* = 160); (b) proven negative LNs in the lateral neck at the final diagnosis (*n* = 120); (c) selective LN removal (*n* = 15); (d) thyroid cancer other than PTC (*n* = 10); (e) metastasis to other organs at the time of diagnosis (*n* = 2); and (f) other malignancies (*n* = 1). Patients with clinically positive level II LNs were excluded from inclusion because level II dissection is mandatory in such cases, making the prediction of level II LN metastasis unnecessary. Finally, we enrolled 517 patients including 355 female (mean age, 41.9 (range, 9–80) years) and 162 male (mean age, 43.1 (range, 6–73) years) patients (Figure 1). 

### 2.2. Image Analysis

US images were obtained using 4–15 MHz (SuperSonic Imagine, Aix-en-Provence, France), 5–12 MHz (iU22; Philips Medical Systems, Best, The Netherlands), and 6–18 MHz (Acuson S2000; Simens Healthcare, Erlangen, Germany) linear array transducers by one of six radiologists with 5–18 years of experience in thyroid imaging. Preoperative US images of both the index cancer and metastatic LNs in the lateral neck were retrospectively reviewed by two experienced radiologists working in consensus. The index cancer was assessed for tumor size (maximum diameter using calipers), shape (parallel: anteroposterior [AP] diameter ≤ transverse diameter in the transverse plane; nonparallel: AP diameter > transverse diameter in the transverse plane), internal composition (solid: no obvious cystic component; mixed: solid with some cystic areas; cystic: predominantly cystic), echogenicity (hyperechoic: hyperechoic relative to the normal thyroid parenchyma; isoechoic: same echogenicity as that of the normal thyroid parenchyma; hypoechoic: hypoechoic relative to the normal thyroid parenchyma and hyperechoic relative to the anterior neck muscles; markedly hypoechoic: hypoechoic or similar echogenicity relative to the anterior neck muscles), margin (well-defined: obviously discernible smooth edges; microlobulated: slightly irregular with small lobulations; irregular: non-smooth edges with spiculations), calcification (no calcification: absence of calcific deposits; macrocalcification: large hyperechoic foci with posterior acoustic shadowing; microcalcification: punctate hyperechoic foci within the solid component of a nodule), tumor location (nonupper, upper: involving the upper 1/3 of the thyroid parenchyma) and extrathyroidal extension (no, yes: abutting or protruding to the capsule or strap muscle). For lateral LNs, the largest LN by dimension was chosen and evaluated for longitudinal diameter, presence or absence of cystic change and calcification, shape (oval: longer axis at least twice the shorter axis; round/irregular: length and width similar or outline not smooth), echogenicity compared to adjacent muscle tissue (hypoechoic/isoechoic: less or same echogenicity; hyperechogenicity: higher echogenicity), presence or absence of the fatty hilum, multilevel involvement (metastasis in more than one cervical LN level), and specific assessment of metastasis in level III LNs [27,28].

### 2.3. Surgery and Pathologic Analysis 

At our institution, we routinely perform total thyroidectomy with a prophylactic central compartment dissection. For patients with LN metastasis confirmed by a preoperative US-guided fine-needle aspiration biopsy or an intraoperative frozen section, we also proceeded with lateral compartment LN dissection. This comprehensive lateral compartment dissection includes levels II, III, IV, and anterior V. One pathologist with 30 years of experience in thyroid pathology assessed the histologic results. Based on pathological reports, we analyzed tumor characteristics such as size, multifocality, bilaterality, extrathyroidal extension, and BRAF mutation status. Additionally, we examined the histological records of the lateral LNs, documenting the largest LN size, total number of harvested LNs, total number of metastatic LNs, LN ratio (metastatic LNs/harvested LNs), and presence of extranodal extension. The TNM stage was determined according to the 7th edition of the American Joint Committee on Cancer staging system.

### 2.4. Statistics

We used the Shapiro–Wilk test to assess the normality of continuous variables, and it showed that none of the variables satisfied the normality assumption. Therefore, we used the nonparametric Mann–Whitney U test for analysis and presented the results as median (IQR). Categorical variables were analyzed using the chi-square test and Fisher’s exact test to assess statistical differences in clinicopathologic and US imaging features between the level II and non-level II metastasis groups. If more than 20% of the cells have an expected frequency of less than 5, Fisher’s exact test was used instead of the Chi-square test. Logistic regression analysis was performed with the stepwise method for variable selection to analyze the features associated with level II LN metastasis, and odds ratios (ORs) and 95% confidence intervals (CIs) were also calculated. Statistical analysis was performed using SAS ver. 9.2 (SAS Institute Inc., Cary, NC, USA). For the correlation between variables, we used the chi-square test (Fisher’s exact test) for categorical–categorical variables. For categorical–continuous variables, we employed the Mann–Whitney U test (for two groups) or the Kruskal–Wallis test (for three or more groups). For continuous–continuous variables, we used Spearman’s rank correlation. The Mann–Whitney U test was conducted using the npar1way procedure, the Chi-square test (Fisher’s exact test) was performed using the freq procedure, and logistic regression was carried out using logistic procedure. Statistical significance was set at *p* < 0.05.

## 3. Results

A total of 517 patients with N1b PTC were analyzed and divided into those without level II LN metastasis (*n* = 321) and those with level II LN metastasis (*n* = 196). The clinicopathologic characteristics (Table 1) revealed no significant differences between the groups in terms of sex (*p* = 0.909) and age (*p* = 0.112). However, patients with level II LN metastasis had a significantly higher proportion of non-conventional PTC pathology (22.4% vs. 10.9%, *p* < 0.001) and a larger median pathologic tumor size (15.0 mm vs. 12.0 mm, *p* < 0.001). A total of 79 non-conventional variant PTC included 65 diffuse-sclerosing variant, 6 follicular variant, 4 tall-cell variant, 1 columnar-cell variant, 1 Hobnail variant, 1 oncocytic variant, and 1 cribriform variant PTC. The level II metastasis group had a higher incidence of multifocality (39.8% vs. 31.2%, *p* = 0.045) and extrathyroidal extension (84.7% vs. 75.1%, *p* = 0.01), while there was no significant difference in TNM stage distribution (*p* = 0.134), bilaterality (*p* = 0.076), and BRAF mutation status (*p* = 0.254). Further analysis of LN characteristics showed that the median pathologic LN size was larger in the level II metastasis group (12.0 mm vs. 9.0 mm, *p* < 0.001), and they had a higher median number of harvested LNs (46.5 vs. 41.0, *p* < 0.001) and metastatic LNs (12.0 vs. 6.0, *p* < 0.001). The LN ratio was also higher in the level II metastasis group (0.3 vs. 0.2, *p* < 0.001).

The imaging characteristics of the tumors and LNs were compared between the groups in Table 2. Tumors in the level II metastasis group were significantly larger (16.0 mm vs. 13.0 mm, *p* < 0.001), and there was a higher prevalence of nonparallel tumors (55.1% vs. 42.4%, *p* = 0.005). There were no significant differences in tumor composition (*p* = 0.679), echogenicity (*p* = 0.674), margin (*p* = 0.169), or calcification (*p* = 0.165) between the groups. Additionally, no significant differences were found in tumor location (upper vs. non-upper, *p* = 0.439) or extrathyroidal extension on imaging (*p* = 0.355). While there were no significant differences in LN cystic change (*p* = 0.443), shape (*p* = 0.345), echogenicity (*p* = 0.950), or presence of hilum (*p* = 0.152), LN calcification was more common in the level II metastasis group (48.5% vs. 37.7%, *p* = 0.016). Multilevel involvement (74.5% vs. 52.0%, *p* < 0.001) and level III involvement (74.0% vs. 53.3%, *p* < 0.001) were significantly higher in the level II metastasis group.

Univariable logistic regression was performed, and the significant variables were used for multivariable logistic regression (Table 3 and Table 4). To consider multicollinearity, we calculated the variance inflation factor (VIF) for each model: US features (preoperative model) and pathologic features (postoperative model). In the US features (preoperative model, Supplementary Table S1), all variables had a VIF of approximately 1.2 or lower, indicating no multicollinearity. However, in the pathologic features (postoperative model, Supplementary Table S1), the LN ratio variable showed a high VIF of 4 or higher due to the strong correlation among the variables calculated as the total number of metastatic LNs/total number of harvested LNs. This suggests the presence of multicollinearity. Therefore, we excluded the LN ratio and the other two correlated variables from the multivariable model.

**Figure 2:**
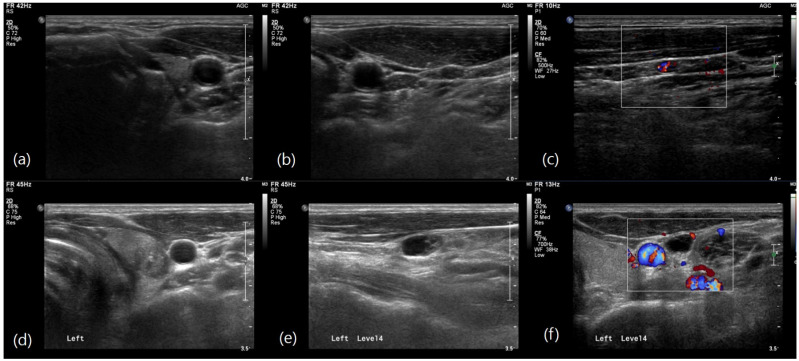
Ultrasound images in N1b papillary thyroid carcinoma. (**a**) Preoperative ultrasound image of a 55-year-old man showing a 0.6-cm, taller-than-wide hypoechoic cancer with microcalcifications in the left upper portion of the thyroid. (**b**,**c**) A 0.5-cm hyperechoic lymph node with increased vascularity is shown on the left side of the neck level III. The final pathology after neck dissection revealed seven metastatic lymph nodes involving level II, III, and IV. (**d**) Preoperative ultrasound image of a 41-year-old man showing a 1-cm, hypoechoic cancer with microcalcifications in the left upper portion of the thyroid. (**e**,**f**) A 1-cm metastatic lymph node with cystic change and peripheral vascularity is shown on the left side of the neck at level IV. The final pathology after neck dissection revealed two metastatic lymph nodes at level IV without level II lymph node involvement.

**Figure 3:**
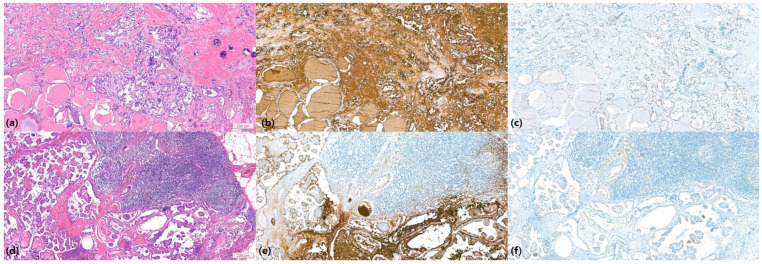
Haematoxylin-Eosin (HE) staining images of the thyroid and metastatic lymph nodes in a 55-year-old man with papillary thyroid carcinoma shown in Figure 2a–c. (**a**) Papillary carcinoma in thyroid (HE staining). (**b**) Cytoplasmic expression of thyroglobulin in papillary thyroid carcinoma by immunohistochemistry. (**c**) Nuclear expression of TTF-1 in papillary thyroid carcinoma by immunohistochemistry. (**d**) Metastatic papillary thyroid carcinoma in a cervical lymph node (HE staining). (**e**) Cytoplasmic expression of thyroglobulin in metastatic carcinoma by immunohistochemistry. (**f**) Focal positivity of TTF-1 in metastatic carcinoma by immunohistochemistry. Scale bar in figures represent 200 μm.

Multivariable logistic regression analysis identified several independent predictors of level II LN metastasis. In the preoperative model (Table 3), larger tumor size (OR, 1.031; 95% CI: 1.011–1.051; *p* = 0.002) and nonparallel tumor shape (OR, 1.963; 95% CI: 1.322–2.915; *p* = 0.001) were significantly associated with level II metastasis. Additionally, the involvement of multiple LN levels and level III were strong predictors with odds ratios of 1.906 (95% CI: 1.242–2.925; *p* = 0.003) and 1.867 (95% CI: 1.223–2.850; *p* = 0.004) respectively. In the postoperative model (Table 4), non-conventional pathology was a significant predictor (OR, 1.951; 95% CI: 1.121–3.396; *p* = 0.018). The presence of extrathyroidal extension (OR, 1.867; 95% CI: 1.060–3.331; *p* = 0.031) and a higher LN ratio (OR, 1.057; 95% CI: 1.039–1.076; *p* < 0.001) were also significantly associated with level II metastasis. These findings highlight the importance of comprehensive preoperative evaluation, including a detailed assessment of tumor size, shape, pathology, and LN characteristics, to identify patients at higher risk of level II LN metastasis (Figure 2 and Figure 3). 

We also analyzed the correlation between the preoperative US features of the index tumor and LN with the pathologic features of the LN (Supplementary Table S2). Pathologic LN size was significantly correlated with tumor size (r = 0.184, *p* < 0.001), total number of metastatic LNs (r = 0.295, *p* < 0.001), and LN ratio (r = 0.308, *p* < 0.001). LN size had a strong positive correlation with pathologic LN size (r = 0.507, *p* < 0.001) and moderate correlations with the total number of metastatic LNs (r = 0.188, *p* < 0.001) and LN ratio (r = 0.164, *p* < 0.001). LN cystic change and LN calcification were significantly associated with pathologic LN size (*p* = 0.001 and *p* < 0.001, respectively). LN shape and echogenicity were also significantly correlated with pathologic LN size (both *p* < 0.001). Additionally, multilevel involvement was significantly associated with pathologic LN size (*p* < 0.001), total number of harvested LNs (*p* < 0.001), total number of metastatic LNs (*p* < 0.001), and LN ratio (*p* < 0.001). Tumor margin, LN echogenicity, and multilevel involvement showed significant differences between cases with and without extranodal extension (*p* = 0.004, *p* < 0.001, and *p* = 0.001, respectively). These findings suggest that specific US features of index tumor and LNs can be predictive of pathologic LN characteristics in a papillary thyroid carcinoma.

## 4. Discussion

The surgical extent of LN dissection in patients with N1b thyroid carcinoma remains unclear. Comprehensive LN dissection can provide almost complete removal of metastatic LNs; however, postoperative morbidity, such as shoulder syndrome, can occur during level II dissection. Therefore, it is important to determine which patients could undergo selective LN excision rather than comprehensive dissection. In this study, we showed that the US features of the index cancer and LNs could predict level II metastasis. In the preoperative model, the tumor size, nonparallel shape of the index tumor, multilevel involvement, and level III involvement of metastatic LNs, were associated with level II LN metastasis. 

Our study showed that a larger tumor size (OR, 1.031; 95% CI: 1.011–1.051; *p* = 0.002) and nonparallel tumor shape (OR, 1.963; 95% CI: 1.322–2.915; *p* = 0.001) were independently associated with level II LN metastasis in a preoperative model (Table 3). Several studies have investigated risk factors for level II metastasis in PTC [10,14,16,20,22,23,24,25,26]. However, there are no data regarding the imaging features of the index cancer and LNs associated with level II LN metastasis. In previous studies, tumor-related risk factors, including capsule invasion and upper location, were associated with level IIb LN metastasis [22]. However, extrathyroidal extension and upper location were not associated with level II LN metastasis in the multivariate analysis in our study. Instead, our study showed that preoperative US features of the index cancer, such as tumor size and nonparallel orientation, were helpful in predicting level II LN metastasis in patients with N1b PTC. A recent study also suggested that tumor size > 20 mm, was associated with level II LN metastasis [10,24]. 

Regarding lateral LNs, location-related features, including multilevel (OR, 1.906; 95% CI: 1.242–2.925; *p* = 0.003) and level III (upper) involvement (OR, 1.867; 95% CI: 1.223–2.850; *p* = 0.004), were associated with level II LN metastasis, but not with the feature of the LN itself (Table 3). These results are consistent with those of previous studies showing that a multilevel or upper-level involvement of LNs was associated with level II LN metastasis [14,16,20,24]. However, our study differs from others in that it specifically excludes patients with preoperative positive level II LN metastasis, focusing on the prediction of occult level II LNs. The current study suggests that a comprehensive LN dissection should be performed when multiple and upper-level involvements are present, even in the absence of a clinically apparent level II LN involvement. 

In the postoperative model (Table 4), non-conventional pathology (OR, 1.951; 95% CI: 1.121–3.396; *p* = 0.018), the presence of an extrathyroidal extension (OR, 1.867; 95% CI: 1.060–3.331; *p* = 0.031), and a higher LN ratio (OR, 1.057; 95% CI: 1.039–1.076; *p* < 0.001) were associated with level II LN metastasis. Non-conventional variants, which include diffuse sclerosing, a tall cell, a hobnail, and columnar cell variants, are often characterized by more aggressive behavior and a propensity for early and extensive metastasis [29]. It suggests that patients with non-conventional pathology should be monitored closely for potential level II LN involvement and may benefit from a more aggressive surgical approach. Extrathyroidal extension is a well-known prognostic factor in thyroid cancer, indicating a more advanced disease and a higher likelihood of LN metastasis [22]. However, in the preoperative setting in our study, the preoperative US feature of extrathyroidal extension did not exert a significant influence, compared to pathological factors. Furthermore, our study revealed that the LN ratio, a known prognostic factor [8], correlated with the presence of level II LN involvement.

Our study highlights significant correlations between preoperative US features of tumors and LNs and their pathologic characteristics, demonstrating that larger tumors and LNs, as well as specific US features such as tumor composition, tumor calcification, LN cystic change, calcification, echogenicity, hilum, multilevel, and level III involvement, are predictive of greater metastatic involvement of LNs (Supplementary Table S2). Notably, the presence of multilevel involvement and differences in tumor margin and LN echogenicity are essential predictors of extranodal extension, a prognostic factor in N1b PTC. These findings support the integration of detailed US evaluation into routine clinical assessments to improve the accuracy of preoperative predictions and influence treatment strategies. 

The greatest significance of this study is that the “preoperative” US features of the index cancer and lateral LN can help determine the surgical methods for the dissection of metastatic LNs in N1b PTC. Unlike the pathologic features of PTC, preoperative US features can be routinely obtained “before” surgery. In addition, they have advantages over other imaging techniques, such as low cost and a lack of contrast agents. Based on the results of our study, a microcarcinoma showing single and low-level LN metastasis can be a good candidate for selective LN removal rather than extensive LN dissection. The potential barriers to adoption of preoperative US, such as variability in operator proficiency in performing and interpreting preoperative US and the need for standardized protocols, pose challenges for future clinical practice.

This study had several limitations. First, because it was conducted retrospectively in a single institution, there may have been a selection bias. The patient population in our study, characterized by high proportions of level II metastasis and extranodal extension due to the aggressive behavior of N1b PTC, might not be representative of the broader population, further contributing to potential selection bias. And due to the relatively small study population, there is a potential for overfitting. Second, while imaging assessment was performed through consensus between two radiologists, the US images were obtained by multiple radiologists, introducing potential operator dependency and variability in image acquisition and interpretation. To address these limitations, future research should consider prospective, multi-center studies to enhance the generalizability of the findings. Additionally, further investigation is needed to evaluate the cost implications of implementing these imaging assessments in routine practice and to understand the impact on patient quality of life and long-term outcomes with selective versus comprehensive LN dissection.

In conclusion, preoperative imaging features of the index cancer and LNs, including tumor size and shape, and multilevel and upper-level LN involvement, are associated with level II LN metastasis. These findings highlight the potential for these imaging features to guide surgical decisions regarding LN dissection in patients with N1b PTC. By incorporating these predictors into clinical practice, we can enhance surgical planning of LN removal in N1 PTC, thus reducing patients’ postoperative morbidities and improving patient outcomes. Furthermore, our study underscores the need for updated clinical guidelines that integrate these imaging characteristics to improve patient outcomes. 

## Figures and Tables

**Table 1:** Clinicopathologic characteristics of patients with N1b papillary thyroid carcinoma according to level II lymph node metastasis.

Characteristics	Level II Metastasis (−) (*n* = 321)	Level II Metastasis (+) (*n* = 196)	*p*-Value
Sex			0.909
Female	221 (68.8)	134 (68.4)	
Male	100 (31.2)	62 (31.6)	
Age (years)			0.112
<55	270 (84.1)	154 (78.6)	
≥55	51 (15.9)	42 (21.4)	
Pathology			<0.001
Conventional	286 (89.1)	152 (77.6)	
Diffuse-sclerosing variant	26 (8.1)	39 (19.9)	
Follicular variant	5 (1.6)	1 (0.5)	
Tall-cell variant	3 (0.9)	1 (0.5)	
Columnar-cell variant	0 (0)	1 (0.5)	
Hobnail variant	0 (0)	1 (0.5)	
Oncocytic variant	1 (0.3)	0 (0)	
Cribriform variant	0 (0)	1 (0.5)	
Pathologic tumor size (mm)	12.0 (8.0–18.0)	15.0 (10.5–22.0)	<0.001
TNM stage			0.134
I	270 (84.1)	154 (78.6)	
II	50 (15.6)	42 (45.7)	
III	1 (0.3)	0 (0.0)	
Multifocality			0.045
No	221 (68.8)	118 (60.2)	
Yes	100 (31.2)	78 (39.8)	
Bilaterality			0.076
No	221 (68.8)	120 (61.2)	
Yes	100 (31.2)	76 (38.8)	
Extrathyroidal extension			0.01
No	80 (24.9)	30 (15.3)	
Yes	241 (75.1)	166 (84.7)	
BRAF status			0.254
No	31 (9.7)	28 (14.3)	
Yes	123 (38.3)	68 (34.7)	
None	167 (52.0)	100 (51.0)	
Pathologic LN size (mm)	9.0 (5.0–14.0)	12.0 (8.0–16.0)	<0.001
Total number of harvested LNs	41.0 (33.0–50.0)	46.5 (35.0–62.0)	<0.001
Total number of metastatic LNs	6.0 (4.0–10.0)	12.0 (7.0–18.0)	<0.001
LN ratio	0.2 (0.1–0.2)	0.3 (0.2–0.4)	<0.001
Extranodal extension			0.105
No	140 (43.6)	67 (34.2)	
Yes	154 (48.0)	110 (56.1)	
None	27 (8.4)	19 (9.7)	

Values are expressed as the median (IQR) or number (%) LN, lymph node; LN ratio, total number of metastatic LNs/total number of harvested LNs.

**Table 2:** Ultrasound features of the index tumor and lateral lymph nodes in patients with N1b papillary thyroid carcinoma according to level II lymph node metastasis.

Characteristics	Level II Metastasis (−) (*n* = 321)	Level II Metastasis (+) (*n* = 196)	*p*-Value
Tumor size	13.0 (9.0–19.0)	16.0 (11.0–24.0)	<0.001
Tumor shape			0.005
Parallel	185 (57.6)	88 (44.9)	
Nonparallel	136 (42.4)	108 (55.1)	
Tumor composition			0.679
Solid	309 (96.3)	186 (94.9)	
<50% cystic	10 (3.1)	8 (4.1)	
>50% cystic	2 (0.6)	2 (1.0)	
Tumor echogenicity			0.674
Hyperechogenicity	1 (0.3)	0 (0.0)	
Isoechogenicity	5 (1.6)	6 (3.1)	
Hypoechogenicity	178 (55.5)	107 (54.6)	
Marked hypoechogenicity	137 (42.7)	83 (42.3)	
Tumor margin			0.169
Well	0 (0)	0 (0)	
Microlobulated	43 (13.4)	35 (17.9)	
Irregular	278 (86.6)	161 (82.1)	
Tumor calcification			0.165
No	91 (28.3)	45 (23.0)	
Macrocalcification	37 (11.5)	17 (8.7)	
Microcalcification	139 (60.1)	134 (68.4)	
Tumor location			0.806
Non-upper	200 (62.3)	120 (61.2)	
Upper	121 (37.7)	76 (38.8)	
Extrathyroidal extension			0.355
No	36 (11.2)	17 (8.7)	
Yes	285 (88.8)	179 (91.3)	
LN size	11.0 (8.0–16.0)	12.0 (9.0–16.0)	0.051
LN cystic change			0.443
No	236 (73.5)	138 (70.4)	
Yes	85 (26.5)	58 (29.6)	
LN calcification			0.016
No	200 (62.3)	101 (59.5)	
Yes	121 (37.7)	95 (48.5)	
LN shape			0.345
Oval	193 (60.1)	126 (64.3)	
Round/irregular	128 (39.9)	70 (35.7)	
LN echogenicity			0.95
Hypo-/isoechogenicity	127 (39.6)	77 (39.3)	
Hyperechogenicity	194 (60.4)	119 (60.7)	
LN hilum			0.152
No	41 (12.8)	17 (8.7)	
Yes	280 (87.2)	179 (91.3)	
Multilevel involvement			<0.001
No	154 (48.0)	50 (25.5)	
Yes	167 (52.0)	146 (74.5)	
Level III involvement			<0.001
No	150 (46.7)	51 (26.0)	
Yes	171 (53.3)	145 (74.0)	

Values are expressed as the median (IQR) or number (%). LN, lymph node.

**Table 3:** Univariate and multivariate logistic regression analysis of preoperative model for predicting level II lymph node metastasis in patients with N1b papillary thyroid carcinoma.

Variables	Univariable		Multivariable Model	
	OR (95% CI)	*p*-Value	OR (95% CI)	*p*-Value
Sex				
Female	ref			
Male	1.024 (0.699–1.502)	0.902		
Age (years)				
<55	ref			
≥55	1.445 (0.918–2.274)	0.112		
Tumor size	1.028 (1.010–1.046)	0.002	1.031 (1.011–1.051)	0.002
Tumor shape				
Parallel	ref		ref	
Nonparallel	1.666 (1.165–2.383)	0.005	1.963 (1.322–2.915)	0.001
Tumor composition				
Solid	ref			
<50% cystic	1.343 (0.521–3.462)	0.541		
>50% cystic	1.660 (0.232–11.888)	0.614		
Tumor echogenicity				
Hyperechogenicity	ref			
Isoechogenicity	3.605 (0.033–395.359)	0.593		
Hypoechogenicity	1.837 (0.019–174.124)	0.793		
Marked hypoechogenicity	1.852 (0.020–175.904)	0.791		
Tumor margin				
Well				
Microlobulated	ref			
Irregular	0.711 (0.437–1.156)	0.169		
Tumor calcification				
No	ref			
Macrocalcification	0.938 (0.478–1.843)	0.854		
Microcalcification	1.398 (0.919–2.126)	0.118		
Tumor location				
Non-upper	ref	0.808		
Upper	1.046 (0.726–1.508)			
Extrathyroidal extension				
No	ref	0.357		
Yes	1.330 (0.725–2.439)			
LN size	1.008 (0.983–1.033)	0.556		
LN cystic change				
No	ref			
Yes	1.168 (0.788–1.733)	0.439		
LN calcification				
No	ref			
Yes	1.553 (1.084–2.225)	0.017		
LN shape				
Oval	ref			
Round/irregular	0.839 (0.581–1.212)	0.350		
LN echogenicity				
Hypo-/isoechogenicity	ref			
Hyperechogenicity	1.011 (0.703–1.454)	0.954		
LN hilum				
No	ref			
Yes	1.518 (0.838–2.749)	0.168		
Multilevel involvement				
No	ref		ref	
Yes	2.676 (1.815–3.945)	<0.001	1.906 (1.242–2.925)	0.003
Level III involvement				
No	ref		ref	
Yes	2.479 (1.684–3.650)	<0.001	1.867 (1.223–2.850)	0.004

LN, lymph node; OR, odds ratio; CI, confidence interval.

**Table 4:** Univariate and multivariate logistic regression analysis of postoperative model for predicting level II lymph node metastasis in patients with N1b papillary thyroid carcinoma.

Variables	Univariable		Multivariable Model	
	OR (95% CI)	*p*-Value	OR (95% CI)	*p*-Value
Pathology				
Conventional	ref			
Non-conventional	2.355 (1.449–3.827)	0.001	1.951 (1.121–3.396)	0.018
Pathologic tumor size (mm)	1.024 (1.006–1.043)	0.009		
TNM stage				
I	ref			
II	1.473 (0.934–2.323)	0.095		
III	0.551 (0.006–54.687)	0.799		
Multifocality				
No	ref			
Yes	1.460 (1.008–2.116)	0.046		
Bilaterality				
No	ref			
Yes	1.399 (0.965–2.029)	0.077		
Extrathyroidal extension				
No	ref			
Yes	1.820 (1.145–2.891)	0.011	1.879 (1.060–3.331)	0.031
BRAF status				
No	ref			
Yes	0.613 (0.340–1.107)	0.105		
None	0.663 (0.376–1.171)	0.157		
Pathologic LN size (mm)	1.045 (1.017–1.075)	0.002		
Total number of harvested LNs	1.019 (1.010–1.029)	<0.001		
Total number of metastatic LNs	1.122 (1.089–1.156)	<0.001		
LN ratio (per 0.01 units)	1.059 (1.043–1.076)	<0.001	1.057 (1.039–1.076)	<0.001
Extranodal extension				
No	ref			
Yes	1.489 (1.018–2.177)	0.040		
None	1.476 (0.767–2.840)	0.244		

LN, lymph node; LN ratio, total number of metastatic LNs/total number of harvested LNs; OR, odds ratio; CI, confidence interval.

## Data Availability

The raw data supporting the conclusions of this article will be made available by the authors on request.

## Supplementary Materials

The following supporting information can be downloaded at: https://www.mdpi.com/article/10.3390/biomedicines12071588/s1, Table S1: Variance Inflation Factor (VIF) for multicollinearity among predictors in the preoperative and the postoperative model; Table S2: The correlation between the preoperative US features of the index tumor and LNs and pathologic features of LNs in N1b PTC.
