# Supplementary material for: Preoperative Ultrasonography Predicts Level II Lymph Node Metastasis in N1b Papillary Thyroid Carcinoma: Implications for Surgical Planning
Source: Biomedicines. 2024 Jul 17;12(7):1588. doi: 10.3390/biomedicines12071588 (PMC11274539; doi:10.3390/biomedicines12071588)
Supplement: Supplementary file 1 [file biomedicines-12-01588-s001.zip › biomedicines-3055088-supplementary.pdf]

Supplementary

Table S1. Variance Inflation Factor (VIF) for multicollinearity among predictors in the preoperative and the postoperative model

| variable | vif |
|---|---|
| Tumor size | 1.127481 |
| Tumor shape | 1.089634 |
| LN calcification | 1.01565 |
| Multilevel involvement | 1.228312 |
| Level III involvement | 1.180165 |

| variable | vif |
|---|---|
| Pathologic tumor size (mm) | 1.20939 |
| Pathologic LN size (mm) | 1.208269 |
| Total number of harvested LNs | 4.303605 |
| Total number of metastatic LNs | 9.416081 |
| LN ratio | 5.8115 |
| Pathology | 1.10641 |
| Multifocality | 1.079336 |
| Extrathyroidal extension | 1.086122 |
| Extranodal extension | 1.065918 |

Table S2. The correlation between the preoperative US features of the index tumor and LNs and pathologic features of LNs in N1b PTC. Cells highlighted in yellow indicate significant values ($p < 0.05$).

| Variables | Pathologic LN size (mm) | | Total number of harvested LNs | | Total number of metastatic LNs | | LN ratio | |
|---|---|---|---|---|---|---|---|---|
| | r(95% CI) or median(IQR) | p-value | r(95% CI) or median(IQR) | p-value | r(95% CI) or median(IQR) | p-value | r(95% CI) or median(IQR) | p-value |
| Tumor size | 0.184(0.092-0.273) | <.001 | 0.080(-0.007-0.165) | 0.070 | 0.295(0.214-0.372) | <.001 | 0.308(0.227-0.383) | <.001 |
| LN size | 0.507(0.433-0.573) | <.001 | 0.090(0.003-0.174) | 0.042 | 0.188(0.103-0.270) | <.001 | 0.164(0.079-0.247) | <.001 |
| Tumor shape | | 0.846 | | 0.393 | | 0.361 | | 0.544 |
| Parallel | 11.0(6.0-15.0) | | 43.0(34.0-56.0) | | 8.0(4.0-14.0) | | 0.2(0.1-0.3) | |
| Nonparallel | 10.0(7.0-15.0) | | 43.0(33.0-53.0) | | 8.0(4.0-12.5) | | 0.2(0.1-0.3) | |
| Tumor composition | | 1.000 | | 0.065 | | 0.030 | | 0.115 |
| Solid | 10.0(6.0-15.0) | | 43.0(34.0-55.0) | | 8.0(4.0-13.0) | | 0.2(0.1-0.3) | |
| <50% cystic | 11.0(8.0-15.0) | | 47.5(36.0-63.0) | | 9.0(7.0-18.0) | | 0.3(0.2-0.3) | |
| >50% cystic | 9.0(8.0-15.0) | | 68.0(49.0-90.0) | | 13.5(11.0-20.0) | | 0.3(0.2-0.3) | |
| Tumor echogenicity | | 0.320 | | 0.209 | | 0.296 | | 0.269 |
| Hyperechogenicity | .(.-.) | | 28.0(28.0-28.0) | | 14.0(14.0-14.0) | | 0.5(0.5-0.5) | |
| Isoechogenicity | 13.0(11.0-15.0) | | 63.0(35.0-78.0) | | 12.0(5.0-28.0) | | 0.3(0.1-0.4) | |
| Hypoechogenicity | 10.0(7.0-15.0) | | 43.0(34.0-56.0) | | 8.0(4.0-13.0) | | 0.2(0.1-0.3) | |
| Marked hypoechogenicity | 10.0(5.0-15.0) | | 42.5(35.0-53.0) | | 8.0(5.0-13.0) | | 0.2(0.1-0.3) | |

| | | | | | | | | |
|---|---|---|---|---|---|---|---|---|
| Tumor margin | | 0.592 | | 0.642 | | 0.622 | | 0.705 |
| Well | - | | - | | - | | - | |
| Microlobulated | 11.0(6.0-16.0) | | 42.5(34.0-53.0) | | 8.0(4.0-14.0) | | 0.2(0.1-0.3) | |
| Irregular | 10.0(6.0-15.0) | | 43.0(34.0-55.0) | | 8.0(4.0-13.0) | | 0.2(0.1-0.3) | |
| Tumor calcification | | 0.059 | | 0.178 | | <.001 | | <.001 |
| No | 9.0(6.0-13.0) | | 41.0(34.5-50.0) | | 7.0(4.0-10.5) | | 0.2(0.1-0.3) | |
| Macrocalcification | 12.0(7.0-17.0) | | 41.0(30.0-57.0) | | 5.5(3.0-10.0) | | 0.2(0.1-0.2) | |
| Microcalcification | 10.5(6.0-16.0) | | 44.0(34.0-57.0) | | 9.0(5.0-15.0) | | 0.2(0.1-0.3) | |
| Tumor location | | 0.163 | | 0.133 | | 0.321 | | 0.855 |
| Non-upper | 11.0(7.0-16.0) | | 43.0(34.0-56.0) | | 8.0(4.0-14.0) | | 0.2(0.1-0.3) | |
| Upper | 10.0(5.0-15.0) | | 43.0(33.0-51.0) | | 7.5(5.0-12.0) | | 0.2(0.1-0.3) | |
| LN cystic change | | 0.001 | | 0.314 | | 0.213 | | 0.442 |
| No | 9.0(5.0-14.0) | | 43.0(33.0-55.0) | | 8.0(4.0-13.0) | | 0.2(0.1-0.3) | |
| Yes | 12.0(7.0-17.0) | | 44.0(36.0-54.0) | | 8.0(5.0-15.0) | | 0.2(0.1-0.3) | |
| LN calcification | | <.001 | | 0.701 | | 0.042 | | 0.038 |
| No | 9.0(5.0-13.0) | | 42.0(35.0-53.0) | | 8.0(4.0-12.0) | | 0.2(0.1-0.3) | |
| Yes | 12.0(8.0-18.0) | | 44.0(33.0-57.0) | | 8.0(5.0-15.0) | | 0.2(0.1-0.3) | |
| LN shape | | <.001 | | 0.275 | | 0.744 | | 0.158 |
| Oval | 10.0(5.0-14.0) | | 42.0(33.0-54.0) | | 8.0(4.0-13.0) | | 0.2(0.1-0.3) | |

| | | | | | | | | |
|---|---|---|---|---|---|---|---|---|
| Round/irregular | 11.5(7.0-18.0) | | 44.0(35.0-55.0) | | 8.0(5.0-13.0) | | 0.2(0.1-0.3) | |
| LN echogenicity | | <.001 | | 0.004 | | 0.010 | | 0.199 |
| Hypo-/isoechogenicity | 7.0(4.0-11.0) | | 41.0(33.0-49.0) | | 7.0(4.0-12.0) | | 0.2(0.1-0.3) | |
| Hyperechogenicity | 13.0(8.0-18.0) | | 45.0(35.0-58.0) | | 8.0(5.0-14.0) | | 0.2(0.1-0.3) | |
| LN hilum | | <.001 | | 0.317 | | 0.011 | | 0.010 |
| No | 5.0(3.0-9.0) | | 40.0(33.0-48.0) | | 6.0(4.0-10.0) | | 0.1(0.1-0.2) | |
| Yes | 11.0(7.0-16.0) | | 43.0(34.0-55.0) | | 8.0(5.0-13.0) | | 0.2(0.1-0.3) | |
| Multilevel involvement | | <.001 | | <.001 | | <.001 | | <.001 |
| No | 8.0(5.0-12.0) | | 40.0(31.0-49.0) | | 5.0(3.0-8.5) | | 0.1(0.1-0.2) | |
| Yes | 12.0(8.0-17.0) | | 45.0(36.0-58.0) | | 10.0(6.0-15.0) | | 0.2(0.2-0.3) | |
| Level III involvement | | <.001 | | 0.004 | | <.001 | | 0.004 |
| No | 9.0(5.0-13.0) | | 41.0(32.0-49.0) | | 7.0(4.0-11.0) | | 0.2(0.1-0.3) | |
| Yes | 11.0(7.0-17.0) | | 44.0(35.0-58.0) | | 8.0(5.0-14.0) | | 0.2(0.1-0.3) | |

| Variables | Extranodal extension | | | |
|---|---|---|---|---|
| | No (n=207) | Yes (n=264) | None (n=46) | p-value |
| | median(IQR) or N% | median(IQR) or N% | median(IQR) or N% | |
| Tumor size | 14.0(10.0-20.0) | 15.0(10.0-22.0) | 13.5(8.0-20.0) | 0.148 |
| LN size | 12.0(9.0-16.0) | 12.0(9.0-17.0) | 10.5(8.0-15.0) | 0.341 |
| Tumor shape | | | | 0.699 |
| Parallel | 105(50.72) | 142(53.79) | 26(56.52) | |
| Nonparallel | 102(49.28) | 122(46.21) | 20(43.48) | |
| Tumor composition | | | | 0.214 |
| Solid | 198(95.65) | 255(96.59) | 42(91.30) | |
| <50% cystic | 6(2.90) | 8(3.03) | 4(8.70) | |
| >50% cystic | 3(1.45) | 1(0.38) | 0(0.00) | |
| Tumor echogenicity | | | | 0.235 |
| Hyperechogenicity | 0(0.00) | 1(0.38) | 0(0.00) | |
| Isoechogenicity | 3(1.45) | 8(3.03) | 0(0.00) | |
| Hypoechogenicity | 126(60.87) | 133(50.38) | 26(56.52) | |
| Marked hypoechogenicity | 78(37.68) | 122(46.21) | 20(43.48) | |
| Tumor margin | | | | 0.004 |
| Well | - | - | - | |
| Microlobulated | 23(11.11) | 41(15.53) | 14(30.43) | |
| Irregular | 184(88.89) | 223(84.47) | 32(69.57) | |
| Tumor calcification | | | | 0.065 |
| No | 57(27.54) | 62(23.48) | 17(36.96) | |
| Macrocalcification | 14(6.76) | 34(12.88) | 6(13.04) | |
| Microcalcification | 136(65.70) | 168(63.64) | 23(50.00) | |
| Tumor location | | | | 0.390 |
| Non-upper | 136(65.70) | 180(68.18) | 35(76.09) | |
| Upper | 71(34.30) | 84(31.82) | 11(23.91) | |
| LN cystic change | | | | 0.165 |
| No | 159(76.81) | 182(68.94) | 33(71.74) | |
| Yes | 48(23.19) | 82(31.06) | 13(28.26) | |
| LN calcification | | | | 0.393 |
| No | 124(59.90) | 147(55.68) | 30(65.22) | |
| Yes | 83(40.10) | 117(44.32) | 16(34.78) | |
| LN shape | | | | 0.129 |
| Oval | 138(66.67) | 152(57.58) | 29(63.04) | |
| Round/irregular | 69(33.33) | 112(42.42) | 17(36.96) | |

| | | | | |
|---|---|---|---|---|
| LN echogenicity | | | | <.001 |
| Hypo-/isoechogenicity | 94(45.41) | 84(31.82) | 26(56.52) | |
| Hyperechogenicity | 113(54.59) | 180(68.18) | 20(43.48) | |
| LN hilum | | | | 0.102 |
| No | 25(12.08) | 24(9.09) | 9(19.57) | |
| Yes | 182(87.92) | 240(90.91) | 37(80.43) | |
| Multilevel involvement | | | | 0.001 |
| No | 97(46.86) | 84(31.82) | 23(50.00) | |
| Yes | 110(53.14) | 180(68.18) | 23(50.00) | |
| Level III involvement | | | | 0.146 |
| No | 88(42.51) | 92(34.85) | 21(45.65) | |
| Yes | 119(57.49) | 172(65.15) | 25(54.35) | |

Values are expressed as the median (IQR) or number (%) LN, lymph node; LN ratio, total number of metastatic LNs/total number of harvested LNs.
